# A dataset on 24-h electrocardiograph, sleep and metabolic function of male type 2 diabetes mellitus

**DOI:** 10.1016/j.dib.2023.109421

**Published:** 2023-07-15

**Authors:** Wenquan Cheng, Hongsen Chen, Leirong Tian, Zhimin Ma, Xingran Cui

**Affiliations:** a Key Laboratory of Child Development and Learning Science, Ministry of Education, School of Biological Sciences & Medical Engineering, Southeast University, Nanjing 210096, China; b Endocrinology Department, Suzhou Science and Technology Town Hospital, Suzhou 21500, China; c Research Center for Learning Science, Southeast University, Nanjing 210096, China

**Keywords:** Electrocardiograph (ECG), Sleep, Metabolic function, T2DM, Glycemic control, Heart rate Variability (HRV)

## Abstract

This dataset provides a collection of 24 h electrocardiograph (ECG) signals, ECG analysis results based on circadian rhythm and R-peak detection, results of sleep quality assessment and clinical indicators of metabolic function acquired from 60 male type 2 diabetes mellitus (T2DM) inpatients. Upon admission, a fasting blood draw and urinary sample were obtained the next morning for routine glucose, lipid and renal panels. Subjects were also involved in investigation for diabetic complications. On the second day of hospitalization, subjects were monitored in hospital for 24-h ECG starting at 10 pm. Subjective sleep quality was assessed by Pittsburgh Sleep Quality Index and a brief sleep log was used to record sleep duration for the studied night. Objective sleep quality and sleep staging were assessed by cardiopulmonary coupling analysis. This dataset could be utilized to conduct conjoint research on the relationships among sleep, metabolic function, and function of cardiovascular system and autonomic nervous system derived from ECG analysis in T2DM, and further investigate the information in ECG signals based on circadian rhythm and physiological status, providing new insights into long term physiological signal processing.

**Specifications Table**

**Table:** 

Subject	Biomedical Engineering
Specific subject area	Physiological signal processing, ECG, sleep, metabolic function
Type of data	Raw data of electrocardiograph (ECG), segmented series of RR-intervals and Tables
How the data were acquired	ECG signals were recorded by ECG recorder AECG-600D. The segmented series of RR-intervals was the analysis results of R-peak detection and sleep staging analysis with the cardiopulmonary coupling (CPC). The table of Objective sleep quality was the analysis results of CPC based on sleep ECG. The table of Subjective sleep quality was the results of Pittsburgh sleep quality index (PSQI). The table of clinical indicators were acquired from routine glucose, lipid and renal tests on the subjects and investigation for diabetic complications.
Data format	Raw Analyzed Filtered
Description of data collection	A total of 60 volunteered inpatients with type 2 diabetes mellitus (T2DM) took part in this study. Exclusion criteria were: female, type 1 diabetes mellitus, history of stroke, subacute myocardial infarction, kidney or liver transplant, other systemic disorders, current recreational drug or alcohol abuse and morbid obesity (body mass index >40).
Data source location	Institution: Suzhou Science & Technology Town Hospital City/Town/Region: Suzhou, Jiangsu Country: China
Data accessibility	Data is hosted on a public repository Repository name: Mendeley Data Data identification number: 10.17632/9c47vwvtss.1 Direct URL to data: https://data.mendeley.com/datasets/9c47vwvtss
Related research article	Cheng W, Chen H, Tian L, Ma Z, Cui X. Heart rate variability in different sleep stages is associated with metabolic function and glycemic control in type 2 diabetes mellitus. Front Physiol. 2023 Apr 14;14:1157270. doi:10.3389/fphys.2023.1157270. PMID: 37123273; PMCID: PMC10140569. [1]

## Value of the Data

• This dataset provides 24 h ECG signals of male T2DM patients during hospitalization, and ECG analysis results based on circadian rhythm and R-peak detection method proposed in our previous study [2]. All ECG signals provided were carefully checked with noise level, artifacts, and ectopic beats. The dataset also includes results of objective and subjective sleep quality assessment and clinical indicators of metabolic function acquired from subjects during hospitalization.
• Researchers, who are interested in the relationships among sleep, metabolic function, and function of cardiovascular system and autonomic nervous system (ANS) derived from ECG analysis in T2DM, can use this dataset to conduct conjoint research.
• This dataset can be used to explore the interaction among sleep, metabolic function and cardiovascular system, and ANS in T2DM patients, to optimize the pre-diagnose and treatment of T2DM and related complications with new analytical methods of long-term physiological signal. For example: 1) the association between physiological function and sleep quality, 2) the impact of circadian rhythm on cardiovascular and ANS function derived from ECG signals, and 3) The alternation of cardiovascular function, HRV derived ANS function and their correlation with metabolic function during sleep cycles.

## 1 Objective

Type 2 diabetes mellitus (T2DM) causes dysfunction of autonomic nervous system (ANS), which brings about significant morbidity and mortality [3]. The ANS plays a crucial role in metabolic regulation, displaying clear circadian rhythm in healthy individuals [4]. Sleep, a vital brain phenomenon, significantly impacts both the ANS and metabolic function [5,6]. In addition to worsening metabolic regulation, the dysfunction of ANS in T2DM patients may also affect circadian rhythms, causing sleep disturbances, further worsening metabolic status and accelerating the progression of type 2 diabetes [7]. Heart rate variability (HRV) is recognized as an effective measure of heart-brain interaction and autonomic activity [8]. However, current research primarily focuses on linear analysis of 24-hour or 5-15 minute electrocardiogram (ECG) recordings obtained in different physiological states without inspection of the underlying autonomic pathways [8]. This approach fails to adequately extract information from long-term signals and overlooks the complex spatial and temporal dynamics and fractal properties of heart rate time series. This dataset provides 24-h ECG signals, ECG analysis results based on circadian rhythm and R-peak detection, and indicators of sleep and metabolic function in T2DM. It can be utilized to conduct conjoint research on the relationships among sleep, metabolic function, and function of cardiovascular system and ANS derived from ECG analysis in T2DM. It can be utilized to further investigate the information in ECG signals based on circadian rhythm and physiological status, providing new insights into long term physiological signal processing.

## 2 Data Description

### 2.1 ECG Signals

ECG recordings were collected by an FDA (U.S. Food and Drug Administration) approved ambulatory single-lead Holter electrocardiogram monitor (DynaDx Corporation, Mountainview, CA, United States), able to record ECG for over 24-h. Sampling frequency of ECG monitoring was set to 250 Hz. The ECG signals were saved in .mat format and were stored in the ECG folder. The variables in the MATLAB structure with corresponding fieldnames are as follows:

• all: ECG during 24-h;
• sleep: ECG during sleep;
• day: ECG during awake;

### 2.2 RR-Interval Time Series

Based on R-peak detection and sleep staging results derived from CPC, the RR interval time sequences during sleep period were segmented according to sleep stages, including unstable sleep, stable sleep and REM sleep. RR interval sequences during the same sleep stage were stitched together as a whole and saved in .mat format. The RR-interval time series files were stored in the RR-interval folder. The variables in the MATLAB structure with corresponding fieldnames are as follows:

• DS: RR-interval time series during unstable sleep;
• RS: RR-interval time series during stable sleep;
• REM: RR-interval time series during REM sleep;

### 2.3 Clinical Indicators of Metabolic Function

During hospitalization, subjects underwent clinical examinations to obtain their metabolic function and assess their physiological status. The results were saved in the Clinical indicators.xlsx excel file. The clinical indicators of metabolic function and diabetic complications statistics are as follows:

• Admission FBG (mmol/L): fasting blood glucose within 24 h of admission;
• Discharge FBG (mmol/L): fasting blood glucose within 24 h before discharge;
• HbA1c (%): glycated hemoglobin;
• SBP (mmHg): systolic blood pressure;
• DBP (mmHg): diastolic blood pressure;
• WBC (× 10^9^/L): white blood cell;
• N% (%): percent of the number of neutrophils in the number of white blood cells;
• Hb (g/L): hemoglobin;
• PLT (× 10^9^/L): platelet;
• CRP (mg/L): c-reactive protein;
• ALT (U/L): alanine aminotransferase;
• AST (U/L): aspartate aminotransferase;
• AST/ALT: ratio of AST to ALT;
• GGT (U/L): gamma glutamyltransferase;
• BUN (mmol/L): blood urea nitrogen;
• UA (mmol/L): uric acid;
• TG (mmol/L): triglycerides;
• HDL-C (mmol/L): high-density lipoprotein-cholesterol;
• LDL-C (mmol/L): low-density lipoprotein-cholesterol;
• UMA (mg): urine micro-albumin;
• UCr (g): urine creatinine;
• UACR (mg/g): urine albumin-creatinine ratio.
• Diabetic Complications: 0/1 means the subject doesn't have/has the complication.
• Diabetic nephropathy: 0/1 means the subject doesn't have/has the complication.
• Diabetic retinopathy and cataract: 0/1 means the subject doesn't have/has the complication.
• Diabetic peripheral neuropathy: 0/1 means the subject doesn't have/has the complication.
• Coronary artery disease and cardiac insufficiency: 0/1 means the subject doesn't have/has the complication.
• Lower extremity atherosclerosis or stenosis: 0/1 means the subject doesn't have/has the complication.
• Carotid plaque: 0/1 means the subject doesn't have/has the complication.

### 2.4 Subjective Sleep Quality

Sleep quality analysis was performed on ECG signals recorded during sleep period of 24 h ECG monitoring. The results of the qualified signal analysis of 38 subjects were saved in the subjective sleep quality.xlsx excel file. The subjective sleep quality was acquired based on a cardiopulmonary coupling analysis algorithm [9]. The sleep quality metrics are as follows:

• AHI (event per hour): apnea-hypopnea index;
• TST (hour): total sleep time;
• UST (hour): unstable sleep time;
• SST (hour): stable sleep time;
• RST (hour): rapid eye movement (REM) sleep time;
• ALUS (min): average length of unstable sleep segments;
• ALSS (min): average length of stable sleep segments;
• ALRS (min): average length of REM sleep segments;
• USP (%): percentage of unstable sleep time in total sleep time;
• SSP (%): percentage of stable sleep time in total sleep time;
• RSP (%): percentage of REM sleep time in total sleep time.

### 2.5 Objective Sleep Quality

Objective sleep quality was assessed by Pittsburgh Sleep Quality Index (PSQI). PSQI includes multiple sleep-related variables over the preceding month, using Likert and open-ended response formats [10]. The PSQI yields seven component scores: subjective sleep quality, sleep latency, sleep duration, habitual sleep efficiency, sleep disturbances, sleep medication, and daytime dysfunction [11]. During 24-h ECG monitoring, 53 subjects filled in PSQI questionnaire and the results were saved in the Objective sleep quality.xlsx excel file.

## 3 Experimental Design, Materials and Methods

### 3.1 Patients

64 volunteered inpatients with T2DM from Suzhou Science & Technology Town Hospital took part in this study. Due to the small number of female inpatients in the study, and to eliminate the interference of perimenopausal syndrome on metabolic status of women in the age group, data from only 60 male inpatients (50 ± 16 years) were included in the follow-up.

### 3.2 Clinical Indicators of Metabolic Function

During hospitalization, subjects underwent clinical examinations to obtain their metabolic function and health status. On admission, fasting blood and urine samples were taken on the following morning for routine glucose, lipid and kidney tests. Subjects also participated in an investigation of diabetic complications.

Subsequently, box plots were used to remove outliers for clinical indicators. Different from other methods that need to assume that the data obey the normal distribution, the drawing of the box graph relies on the actual data, and does not need to assume that the data obey some specific distribution form in advance, which can represent and consider the original appearance of the data shape more truly and intuitively. Meanwhile, the box chart is based on quartile and interquartile distance to judge outliers. The quartile has a certain resistance, and the proportion of outliers is small, which is difficult to affect the quartile. Therefore, for RR-interval time series, the box chart can be used to eliminate outliers. The difference between the upper quartile U and the lower quartile L of the data was defined as the IQR. The upper bound was set to U+1.5IQR and the lower bound was set to L-1.5IQR. Data exceeding the upper and lower bounds can be considered as outliers. However, in order to retain more information under the pathological data, we retained some data that did not appear to be seriously wrong, that is, outside the range of normal values, but may be present in patients with dysfunctional states.

### 3.3 ECG Data Recording

ECG recordings were collected by an FDA (U.S. Food and Drug Administration) approved ambulatory single-lead Holter electrocardiogram monitor (DynaDx Corporation, Mountainview, CA, USA). Sampling frequency of ECG monitoring was set to 250 Hz. Monitoring on ECG signals of all subjects started at 10 pm on the second day of hospitalization, lasting for 24-h. All ECG recordings were checked for noise levels, artifacts, and ectopic rhythms. Seventeen ECG recordings were discarded due to low data quality, overshort recording time, or the presence of atrial fibrillation or server arrhythmias in hospitalized patients.

### 3.4 ECG Data Preprocessing

The accurate detection of R-peak is important for heart rate variability analysis, beat segmentation and arrhythmia recognition. There are many influencing factors during the process, including noise interference and the similarity of characteristics (slope, amplitude and frequency) between T wave and R wave, which cause great interference to R wave detection. Numerous R-peak detection methods have been proposed for analyzing ECG signals. However, when these methods are applied to long-term recorded ECG signals, particularly using wearable single-lead ECG devices, the accuracy of R-peak detection is often unsatisfactory. To improve the accuracy of R-peak detection [2], we employed a method for extracting high-quality RR intervals as proposed in our previous study [2], which combines five common R-peak detection methods [7], [8], [9], [10], [11], and is based on template matching and weighted fusion methods.

The specific steps for calculating the initial R-peak labeling scores are as follows:

(a) Five sets of initial R-peak detection results were obtained by five detection methods [7], [8], [9], [10], [11].
(b) The ECG samples were classified by template matching, and weights were selected according to the categories. There are five categories in total, corresponding to five sets of weights, which includes five weights corresponding to the five detection methods [7], [8], [9], [10], [11]. Method with better performance on specific type of ECG is assigned more weights.
(c) Based on the weights in b), a “score” is calculated for each R-peak labeled in each of the five sets of results in a). Higher score indicates higher accuracy of the R-peak detection.
(d) Combine the five sets of R-peak detection results into a vector and calculate the scores of the initial R-peak annotations obtained in step a) one by one.

### 3.5 Sleep Quality Assessment

Subjective sleep quality was evaluated using the Pittsburgh Sleep Quality Index (PSQI). The assessment utilized Likert and open-ended response formats [5]. Among the 53 subjects who underwent 24-hour ECG monitoring, the PSQI questionnaire was completed.

Objective sleep quality and sleep staging were determined through ECG-based cardiopulmonary coupling (CPC) analysis [4]. This analysis involves mathematical analysis of the combination between heart rate variability and the beat-to-beat respirational modulation of the QRS waveform. The CPC analysis was applied on extracted ECG recordings obtained during sleep. The determination of sleep period was derived from a brief sleep log collected from subjects, which recorded the sleep duration on the night of ECG recording. The CPC analysis categorizes major physiological sleep states into stable sleep, unstable sleep, and rapid eye movement (REM) states [4]. 38 CPC sleep reports were available for analysis in this study.

## Ethics Statements

All participants were informed the experimental protocol and matters needing attention, then signed the written informed consent prior to participating in the study. This study was approved by the Institutional Review Board of Suzhou Science & Technology Town Hospital (No. IRB2019045).

## CRediT authorship contribution statement

**Wenquan Cheng:** Formal analysis, Data curation, Writing – original draft. **Hongsen Chen:** Formal analysis, Data curation. **Leirong Tian:** Software, Validation, Formal analysis. **Zhimin Ma:** Methodology, Formal analysis, Writing – review & editing, Supervision. **Xingran Cui:** Conceptualization, Methodology, Validation, Formal analysis, Writing – review & editing, Supervision, Project administration, Funding acquisition.

## Declaration of Competing Interest

The authors declare that they have no known competing financial interests or personal relationships that could have appeared to influence the work reported in this paper.

## Data Availability

Dataset on electrocardiograph, sleep and metabolic function of male type 2 diabetes mellitus (Original data) (Mendeley Data).

## Appendix Supplementary materials

Image, application 1
