# Supplementary material for: A dataset on 24-h electrocardiograph, sleep and metabolic function of male type 2 diabetes mellitus
Source: Data Brief. 2023 Jul 15;49:109421. doi: 10.1016/j.dib.2023.109421 (PMC10405204; doi:10.1016/j.dib.2023.109421)
Supplement: Supplementary file 1 [file mmc1.doc]

**知情同意书**

**(一) 知情同意书·知情告知页**

亲爱的患者:

我们将邀请您参加一项“2型糖尿病患者睡眠和24小时心率变异性动态变化规律研究”。

在您决定是否参加这项研究之前，请尽可能仔细阅读以下内容，它可以帮

助您了解该项研究以及为何要进行这项研究：

1、研究背景和研究目的

我院伦理委员会己经审议此项研究是遵从赫尔辛基宣言原则，符合医疗道德的。

本研究的目的是研究糖尿病患者的心率变异性动态变化与昼夜节律和代谢调控间的联系，并进一步揭示睡眠对患者自主神经系统功能、代谢功能、血糖控制和相关并发症的影响。其研究结果将用于发表科学论文。

本研究将在苏州科技城医院进行，预计有100名受试者自愿参加。

2、测量设备的原理

本研究将采用医用级可穿戴单导联心电设备和（或）华为手环。所用的心电设备的原理跟目前临床上常规使用的动态心电图机原理相同。本研究所记录的心电信号为单导联心电信号。本研究利用心电设备将测量电极（凝胶电极）放置在人体体表（胸部）记录心脏每一心动周期所产生的电活动变化曲线，进而连续24小时记录其心电活动的全过程。

3、测量具体流程

a) 测量时间为入院后第二天和出院前一天，每次24小时；

b) 测量时为您佩戴心电贴，手环和脉搏血氧仪（可选项），放置BCG测量设备（可选项）；次日早晨摘下脉搏血氧仪，24小时后摘下心电贴和手环；

c) 佩戴仪器期间不能洗澡；

d) 测量结束后您需要填写匹兹堡睡眠质量指数量表（PSQI），以便了解您一个月内的睡眠情况；

4、参加研究可能的受益

您和社会将可能从本项研究中受益。此种受益包括睡眠监护报告，150元的酬金（可选，须佩戴夜间血氧仪及手环），同时您将在研究期间获得良好的医疗服务。

5、参加研究可能的不良反应、风险和不适、不方便

本研究使用医疗级设备和华为手环，无侵入性，不会导致任何严重不良反应。 如果在研究中您出现任何不适，可以随时停止试验。

6、有关费用

本次研究将免费为您进行心电监测和睡眠监测，不会造成任何额外开支。

7、个人信息是保密的吗?

您的医疗记录将完整地保存在医院。研究者、申办者代表、伦理委员会和药品监督管理部门将被允许查阅您的医疗记录。任何有关本项研究结果的公开报告将不会披露您的个人身份。我们将在法律允许的范围内，尽一切努力保护您个人医疗资料的隐私。

**(二) 同意签字页**

临床研究项目名称: 2型糖尿病患者睡眠和24小时心率变异性动态变化规律研究

申办者:苏州科技城医院

伦理审查批件号: IRB2019045

同意声明

我己经阅读了上述有关本研究的介绍，而且有机会就此项研究与医生讨论并提出问题。我提出的所有问题都得到了满意的答复。

我知道参加本研究可能产生的风险和受益。我知晓参加研究是自愿的，

我确认已有充足时间对此进行考虑，而且明白:

· 我可以随时向医生咨询更多的信息。

· 我可以随时退出本研究，而不会受到歧视或报复，医疗待遇与权益不会受到影响。

我同样清楚，如果我中途退出研究，我若将病情变化告诉医生，完成相应的体格检查和理化检查，这将对我本人和整个研究十分有利。

我同意伦理委员会或申办者代表查阅我的研究资料。

最后， 我决定同意参加本项研究，并尽量遵从医嘱。

患者签名: 患者授权委托人签名（与患者关系）：

日期: 日期：

研究人员声明：

我确认己向患者解释了本试验的详细情况，包括其权力以及可能的受益

和风险，并给其一份签署过的知情同意书副本。

研究人员签名:

日期:

研究人员的电子邮箱:xingxm@sibet.ac.cn
